# Identification of candidate genes controlling oil content by combination of genome-wide association and transcriptome analysis in the oilseed crop *Brassica napus*

**DOI:** 10.1186/s13068-019-1557-x

**Published:** 2019-09-10

**Authors:** Zhongchun Xiao, Chao Zhang, Fang Tang, Bo Yang, Liyuan Zhang, Jingsen Liu, Qiang Huo, Shufeng Wang, Shengting Li, Lijuan Wei, Hai Du, Cunmin Qu, Kun Lu, Jiana Li, Nannan Li

**Affiliations:** 1 Chongqing Engineering Research Center for Rapeseed, College of Agronomy and Biotechnology, Southwest University, Chongqing, 400715 China; 2 Research Center of Bioenergy and Bioremediation, College of Resources and Environment, Southwest University, Chongqing, 400715 China; 3 Academy of Agricultural Sciences, Southwest University, Beibei, Chongqing, 400715 China

**Keywords:** *Brassica napus*, GWAS, Seed oil content, SNPs, Transcriptomics, Candidate genes

## Abstract

**Background:**

Increasing seed oil content is one of the most important targets for rapeseed (*Brassica napus*) breeding. However, genetic mechanisms of mature seed oil content in *Brassica napus* (*B. napus*) remain little known. To identify oil content-related genes, a genome-wide association study (GWAS) was performed using 588 accessions.

**Results:**

High-throughput genome resequencing resulted in 385,692 high-quality single nucleotide polymorphism (SNPs) with a minor allele frequency (MAF) > 0.05. We identified 17 loci that were significantly associated with seed oil content, among which 12 SNPs were distributed on the A3 (11 loci) and A1 (one loci) chromosomes, and five novel significant SNPs on the C5 (one loci) and C7 (four loci) chromosomes, respectively. Subsequently, we characterized differentially expressed genes (DEGs) between the seeds and silique pericarps on main florescences and primary branches of extremely high- and low-oil content accessions (HO and LO). A total of 64 lipid metabolism-related DEGs were identified, 14 of which are involved in triacylglycerols (TAGs) biosynthesis and assembly. Additionally, we analyzed differences in transcription levels of key genes involved in de novo fatty acid biosynthesis in the plastid, TAGs assembly and lipid droplet packaging in the endoplasmic reticulum (ER) between high- and low-oil content *B. napus* accessions.

**Conclusions:**

The combination of GWAS and transcriptome analyses revealed seven candidate genes located within the confidence intervals of significant SNPs. Current findings provide valuable information for facilitating marker-based breeding for higher seed oil content in *B. napus.*

## Background

*Brassica napus* (*B. napus*, AACC, 2*n* = 38) is now the second largest oil crop following soybean. Moreover, rapeseed oil is not only one of the major edible vegetable oil for human consumption but it is also important for biofuel and lubricant production for industry [43]. *B. napus* is also a valuable animal feed source and potential protein source for human nutrition owing to the high-quality protein and low glucosinolate content of the seed [29, 53]. Enhancing seed oil content (SOC) and oil production per unit area of land is of paramount importance to meet the growing demand in oilseed breeding programs [61]. Although a comprehensive overview of the biological and metabolic pathways for triacylglycerol (TAG) synthesis has been well recorded [3, 34], little is known about the genetic and complex molecular regulatory mechanisms underlying variations in SOC of *B. napus*.

Quantitative trait locus (QTL) mapping and genome-wide association study (GWAS) have been widely used to dissect the regulatory loci and genetic architecture of complex agronomical quantitative traits at the whole genome level. Previous studies have focused on QTL identification for oil content in *B. napus* and have identified numerous QTLs in all 19 linkage groups of *B. napus* [8, 10, 19, 24, 49, 51, 54, 60]. GWAS, as another alternative for identifying QTLs, is not restricted to the traditional biparental linkage mapping and offers a higher resolution. Additionally, GWAS has recently been widely used in the study of important complex traits in *B. napus*, such as seed germination and vigor [16], plant height and primary branch [32, 52], harvest index [38, 42], yield traits [39, 57]. In addition, GWAS has been reported in various plants, such as *Arabidopsis* [1], rice [21, 22, 65], soybean [23] and maize [28, 50] and so on.

*Brassica napus* is an allopolyploid species with a complex genome structure, which originated ~ 7500 years ago from a spontaneous hybridization between *B. rapa* (AA, 2*n* = 20) and *B. oleracea* (CC, 2*n* = 18) [7]. SOC is an important complex quantitative traits but its genetic and molecular mechanisms remain undefined. So far, there are relatively few reports on the study of SOC by GWAS. Liu et al. identified 50 loci that were significantly associated with SOC using 521 *B. napus* accessions genotyped with the *Brassica* 60 K SNP array by GWAS and validated a novel locus on chromosome A5 that could increase 1.5–1.7% of the seed oil content by linkage mapping [36]. Li et al. detected a QTL on chromosome A08 with a significant association with seed oil content using GWAS [30]. Wang et al. detected 17 loci associated with seed oil content by GWAS [56] using a total of 238 rapeseed cultivars. In this study, we selected 588 *B. napus* accessions for GWAS by high-throughput genome resequencing.

To understand the genetic control of SOC at the population level through the identification of associated loci with SNPs, we genotyped 588 *B. napus* accessions that were collected from Asia (466), Europe (102), North America (13) and Australia (7) using high-throughput genome resequencing, and carried out a GWAS with PCA + K statistical models. SNPs that were significantly associated with SOC were identified. In addition, we performed transcriptome sequencing of four tissues with extremely high-(HO) and low-oil content (LO) *B. napus* accessions. Among the genes identified in both the GWAS and transcriptome analysis, seven were identified as candidate genes involved in seed oil accumulation, which were verified by quantitative real-time PCR (qRT-PCR). The current study thus may contribute to marker-based breeding for higher seed oil content in *B. napus*.

## Materials and methods

### Plant materials and phenotyping

A total of 588 *B. napus* lines were collected from spring, winter and semi-winter accessions and cultivated in Southwest University of Beibei, Chongqing, China (29°45′N, 106°22′E, 238.57 m) for 3 consecutive years (2016–2018). All the field experiments followed a randomized complete block design with two biological replications. Each accession was planted in two rows of 10–12 plants per row, with 20 cm between plants within each row and 30 cm between rows. The trial management was performed in accordance with local standard breeding field protocols. At maturity, open-pollinated seeds including five representative plants in the middle of each plot were collected for the SOC measurements. The oil content of the desiccated seeds was measured by near-infrared reflectance spectroscopy (NIRS DS2500) using the parameters described by Gan et al. [14].

### Genome-wide association analysis

The seed oil content with two biological replicates for 3 consecutive years (2016–2018) was evaluated by the method of the best linear unbiased prediction (BLUP) based on a linear model using an R script (http://www.eXtension.org/pages/61006). An association analysis was implemented in TASSEL5.2.1 software using the P + K model [6]. The population structure (Q), relative kinship (K) and SNP genotyping in the association panel has been well described in our previous report [41]. The uniform threshold of GWAS was *P* < 2.59 × 10^−6^ (1/valid SNPs used, − log_10_ (1/385,692) = 5.59) [56]. The quantile–quantile plot was shown with the expected *P* value and − log 10 (*P*) of each SNP, and the Manhattan plot was demonstrated using the R package qqman.

### Transcriptome sequencing and identification of differentially expressed genes

Two extremely high-oil content (HO) lines and one extremely low-oil content (LO) line were selected from the GWAS population for transcriptome sequencing (RNA-Seq), respectively. The HO lines were SWU47 (CQ24) and Zhongshuang11 (CQ52), while the LO line was Ningyou12 (CQ46). Total RNA was extracted from four tissues of the HO and LO accessions, respectively. Tissues were harvested 30 days after flowering from seed and silique pericarps on the main inflorescence (30SM and 30SPM, respectively) and on the primary branch (30SB and 30SPB, respectively). For each sample, two biological replicates were performed, with each collected from three independent plants. All samples were immediately placed in liquid nitrogen and stored at − 80 °C for RNA sequencing (RNA-seq) and quantitative reverse-transcription polymerase chain reaction (qRT-PCR) analysis.

Sequencing library preparation and sequencing reactions were conducted at the Biomarker Technologies Corporation (Beijing, China). Gene expression levels were estimated using FPKM (Fragments per kilobase of exon per million reads mapped). Differentially expressed genes (DEGs) between two samples were obtained with Cuffdiff, based on the criteria false discovery rate (FDR) < 0.05 and |log_2_fold change| > 2.0 [4].

### Identification and expression analysis of acyl-lipid metabolism genes between extremely high- and low-oil content *B. napus* lines

To seek out genes associated with acyl-lipid metabolism (ALM), *B. napus* homologous gene sequences were analyzed against a list of genes involved in acyl-lipid metabolism obtained from the “Arabidopsis Acyl-Lipid Metabolism” website (ARALIP) (http://aralip.plantbiology.msu.edu/) [34]. The differentially expressed lipid metabolism-related genes were obtained on the basis of the RNA-Seq data of the *B. napus* HO and LO lines created in the present study. Similarly, to analyze the spatial expressions of differentially expressed acyl-lipid metabolism-related genes in the extremely HO and LO *B. napus* lines, the expression values of these genes in 30SM, 30SPM, 30SB and 30SPB were obtained from this RNA-Seq data as well. The expression heatmap of the differential expressed acyl-lipid metabolism genes was generated using HemI1.0 [58].

### GO and KEGG enrichment analysis of DEGs

GO enrichment and KEGG pathway analysis of DEGs were performed using the online OmicShare tool (http://www.omicshare.com/tools/index.php/) [37, 45]. The threshold of significantly enriched GO terms was set to FDR < 0.05 [39].

### Identification of potential candidate genes

To identify candidate genes associated with SOC, the 300-kb flanking regions on either side of the markers significantly associated with SOC were chosen as the confidence interval for in-depth analysis, as described previously [56]. The DEGs within the confidence interval of SNPs significantly associated with SOC were screened to identify differentially expressed (DE) candidate genes. Additionally, DEGs involved in acyl-lipid metabolism were also identified.

### Validation of candidate genes by qRT-PCR analysis

Total RNA was extracted from all tested tissues with the EZ-10 DNAaway RNA Mini-prep Kit [Sangon Biotech (Shanghai), Co., Ltd], and then cDNA was synthesized from 1 µg RNA using the PrimeScript™ RT reagent kit with gDNA Eraser according to the manufacturer’s instructions (Perfect Real Time; TaKaRa Biotechnology, Dalian, China). The gene-specific primers for qRT-PCR of the candidate genes and reference gene are listed in Additional file 1: Table S7. The PCR consisted of 10 μL SYBR II (TakaRa), 2.0 μL cDNA, 1.6 μL primer, 0.4 μL ROX Reference Dye II and distilled water to a final volume of 20 μL. The PCR program was as follows: 95 °C for 30 s and 35 cycles of 95 °C for 5 s, followed by 56–60 °C (depending on the primers used) for 30 s. For each reaction, three biological replicates were performed, and relative expression levels were obtained using the 2^−∆∆Ct^ method, with BnActin7 as internal controls.

## Results

### Phenotypic variation of SOC

**Fig. 1:**
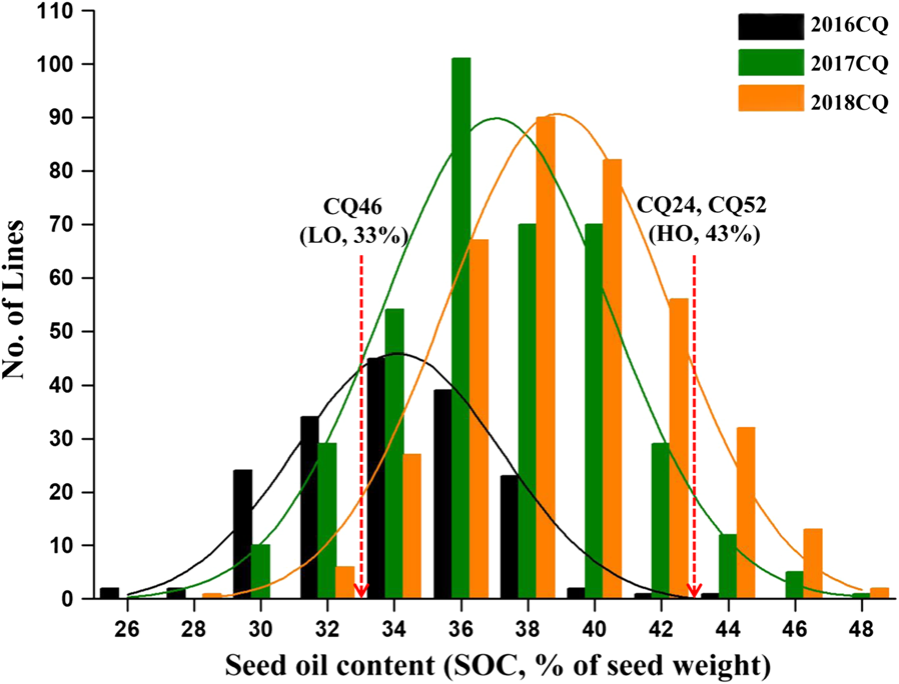
Frequency distribution of seed oil content (SOC) in the association panel of 588 accessions across three environments (2016CQ, 2017CQ and 2018CQ). CQ refers to Chongqing and the No. of Lines represents the number of accessions. CQ46 (Ningyou 12), CQ24 (SWU 47) and CQ52 (Zhongshuang 11) are accessions for RNA-Seq. LO and HO refers to extremely high- and low-oil content *B. napus* accessions, respectively

The 588 *B. napus* accessions were planted in three environments (2016CQ, 2017CQ and 2018CQ) from 2016 to 2018, with two replications performed each year. Extensive phenotypic variations of SOC were observed (Table 1), and specific seed oil content (SOC, % of seed weight) phenotypes of 588 accessions for GWAS analysis are shown in Additional file 1: Table S1. In 2016CQ, SOCs ranged from 26.83 to 44.94, with an average of 35.05. And SOCs were from 30.21 to 48.41, with an average of 38.21 in 2017CQ, and from 29.46 to 49.13, with an average of 40.15 in 2018CQ. Among the three environments, the coefficient of variation (CV) of SOC was less than 10%, reflecting a relatively small variation of SOC within the entire GWAS panels. SOC among three environments in *B. napus* showed continuous variation and approximated a normal distribution (Fig. 1), suggesting SOC consisted of quantitative traits controlled by multiple genes. The broad sense heritability of SOC was 73.4%, which is less than previously reported by Liu et al. [36] (87.4%). These results suggested that most of the phenotypic variation in SOC was attributed to genetic effects despite being greatly affected by the environment in this study.

**Table 1:** Phenotypic variation of seed oil content in three environments

Trait	Mean	SD	Min	Max	CV (%)
2016CQ	35.05	2.88	26.83	44.94	8.22
2017CQ	38.21	3.31	30.21	48.41	8.66
2018CQ	40.15	3.18	29.46	49.13	7.92

*CQ* Chongqing, *SD* standard deviation, *CV* coefficient of variation

### Genome-wide association analysis

**Fig. 2:**
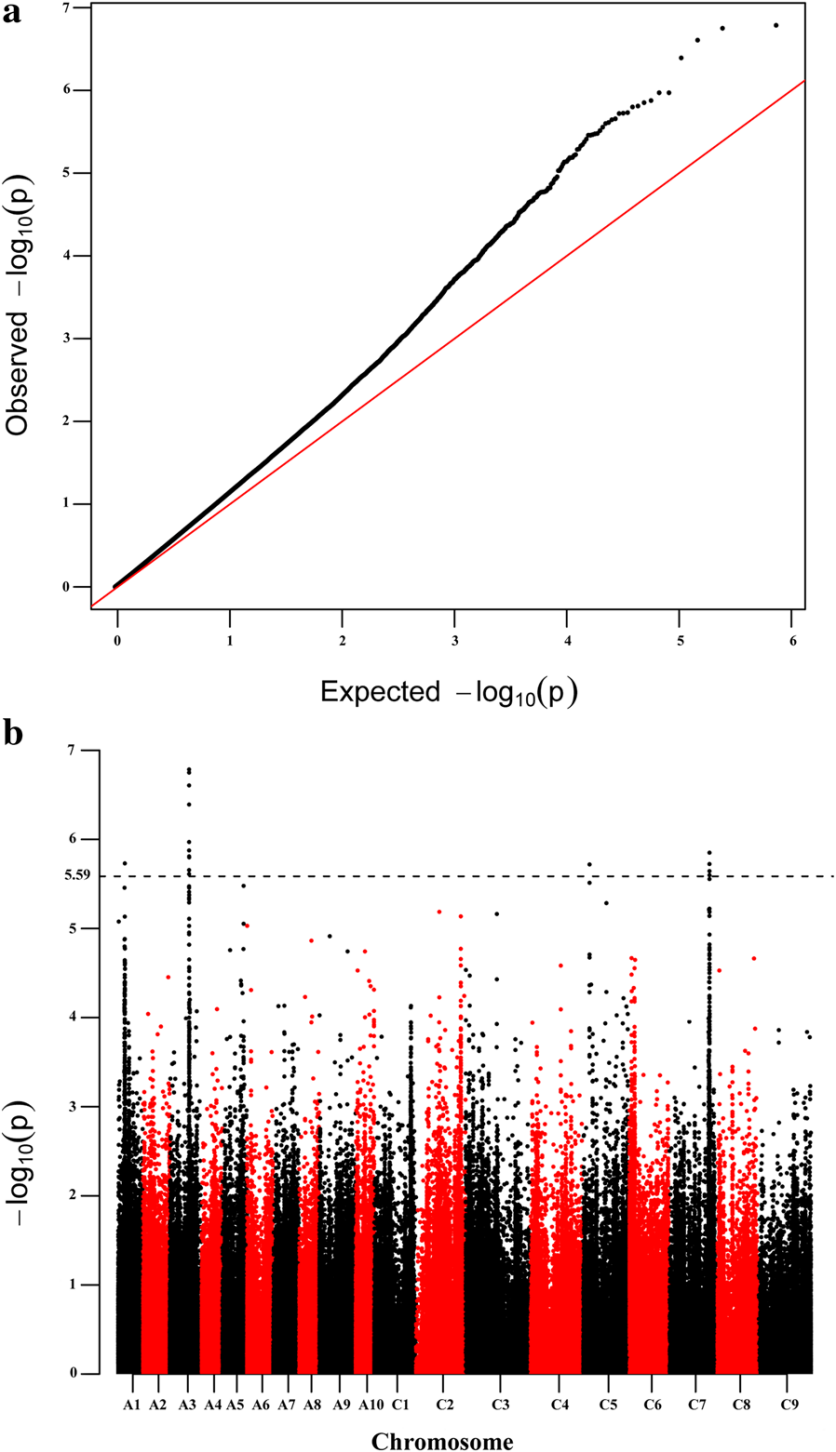
Quantile–quantile and Manhattan plots resulting from the GWAS results for seed oil content (SOC) in *Brassica napus*. **a** Quantile–quantile plot for SOC. **b** Manhattan plot for SOC. The dashed horizontal line indicates the Bonferroni-adjusted significance threshold (2.59 × 10^−6^)

**Fig. 3:**
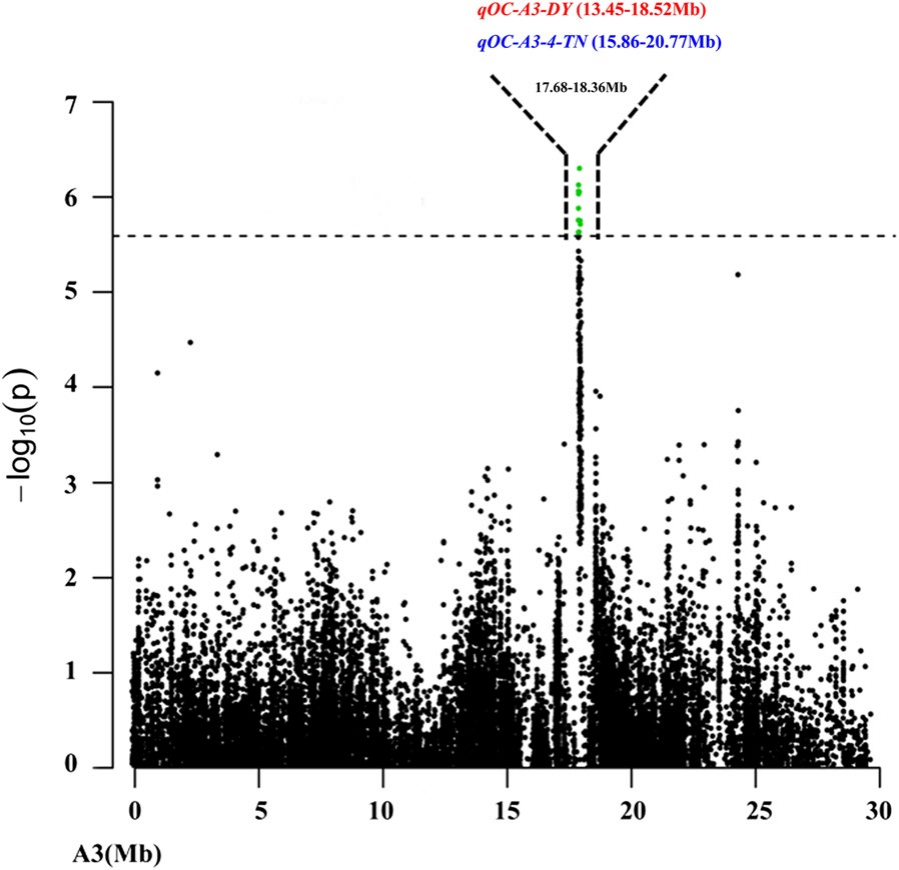
Comparison of the GWAS results in A3 with those of previous QTL mapping reported in Delourme et al. [10] and Zou et al. [68]. The markers that lie within the confidence interval of the seed oil content (SOC) QTL are in red for Delourme et al. [10] and blue for Zou et al. [68]

GWAS for seed oil content (SOC) was performed using the P + K model [48]. The QQ plot is shown in Fig. 2a, and the results showed that this model could be used to identify association SNPs. A total of 17 significant SNPs for SOC (*P* < 2.59 × 10^−6^) were identified and these SNPs were unevenly distributed across four chromosomes (A1, A3, C5 and C7) (Fig. 2b and Table 2). Eleven significant SNPs were distributed on the A3 chromosome and for up to 64.71%, the significant correlation region ranged from 17.68 18.36 Mb, which was consistent with some previous QTL mapping results (Fig. 3) [10, 68]. Only one SNP was distributed on the A1 and C5 chromosomes, respectively. Additionally, the remaining four SNPs were distributed on the C7 chromosome. Individual significant SNPs for SOC explained 5.46–6.68% of the phenotypic variation (*R*^2^). The location and detailed information for all 17 SNPs are listed in Table 2. Among all the detected significant SNPs, twelve SNPs (70.59%) were within the previously identified significant QTL confidence intervals associated with seed oil content, suggesting the high reliability of SNPs identified in this study (Table 2) [10, 36, 54, 66, 68]. To find candidate genes associated with oil content, all these significant SNPs will be further analyzed.

**Table 2:** Markers with significant association for SOC and genes in confidence interval

Chromosome	Position	SNP	*P* value	*R* ^2^	Confidence interval (300 kb up/downstream)	Genes in confidence interval	Detected in previous studies
A1	6244998	S1_6244998	1.86E−06	0.06489	5944998–6,544,998	BnaA01g11880D–BnaA01g13060D	SG-qOC-A1 [66]; qOC-A1-1 [54]; Bn-A01-p6560270 [36]; cqOC-A1 [8]
A3	17975486	S3_17975486	1.64E−07	0.06679	17,675,486–18,275,486	BnaA03g36150D–BnaA03g37050D	qOC-A3-DY, qOC-A3-4-TN [10, 68]
A3	17981854	S3_17981854	1.78E−07	0.06494	17,681,854–18,281,854	BnaA03g36170D–BnaA03g37070D	qOC-A3-DY, qOC-A3-4-TN [10, 68]
A3	17982022	S3_17982022	2.48E−07	0.06503	17,682,022–18,282,022	BnaA03g36170D–BnaA03g37070D	qOC-A3-DY, qOC-A3-4-TN [10, 68]
A3	17981872	S3_17981872	4.06E−07	0.06133	17,681,872–18,281,872	BnaA03g36170D–BnaA03g37070D	qOC-A3-DY, qOC-A3-4-TN [10, 68]
A3	17976024	S3_17976024	1.07E−06	0.05832	17,676,024–18,276,024	BnaA03g36160D–BnaA03g37050D	qOC-A3-DY, qOC-A3-4-TN [10, 68]
A3	17982457	S3_17982457	1.07E−06	0.0598	17,682,457–18,282,457	BnaA03g36170D–BnaA03g37070D	qOC-A3-DY, qOC-A3-4-TN [10, 68]
A3	17986408	S3_17986408	1.33E−06	0.05819	17,686,408–18,286,408	BnaA03g36180D–BnaA03g37080D	qOC-A3-DY, qOC-A3-4-TN [10, 68]
A3	18016213	S3_18016213	1.55E−06	0.05585	17,716,213–18,316,213	BnaA03g36240D–BnaA03g37110D	qOC-A3-DY, qOC-A3-4-TN [10, 68]
A3	18055410	S3_18055410	1.59E−06	0.05956	17,755,410–18,355,410	BnaA03g36320D–BnaA03g37130D	qOC-A3-DY, qOC-A3-4-TN [10, 68]
A3	17986377	S3_17986377	2.21E−06	0.05862	17,686,377–18,286,377	BnaA03g36180D–BnaA03g37080D	qOC-A3-DY, qOC-A3-4-TN [10, 68]
A3	17982838	S3_17982838	2.45E−06	0.05525	17,682,838–18,282,838	BnaA03g36170D–BnaA03g37070D	qOC-A3-DY, qOC-A3-4-TN [10, 68]
C5	5886965	S15_5886965	1.91E−06	0.0638	5,586,965–6,186,965	BnaC05g10000D–BnaC05g10760D	
C7	37164777	S17_37164777	1.41E−06	0.05992	36,864,777–37,464,777	BnaC07g33910D–BnaC07g34940D	
C7	37136168	S17_37136168	1.89E−06	0.05761	36,836,168–37,436,168	BnaC07g33820D–BnaC07g34890D	
C7	37142657	S17_37142657	2.27E−06	0.05463	36,842,657–37,442,657	BnaC07g33850D–BnaC07g34890D	
C7	37178443	S17_37178443	2.52E−06	0.05577	36,878,443–37,478,443	BnaC07g33910D–BnaC07g34970D

### Comparative analysis of three *Brassica napus* lines using transcriptome sequencing

**Fig. 4:**
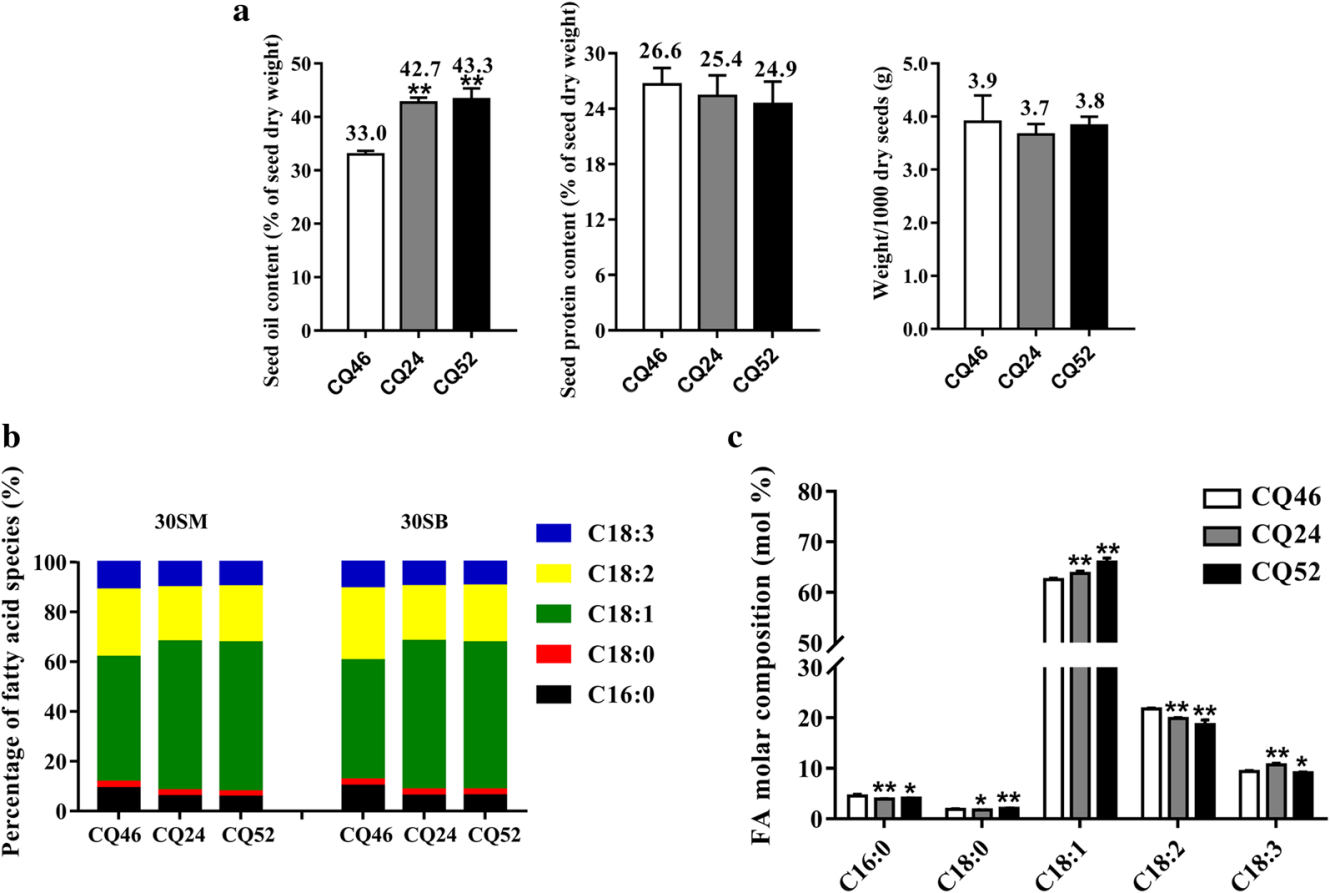
Seed oil content, protein content and fatty acid (FA) composition of HO and LO *Brassica napus* accessions for RNA-Seq. **a** Three years (2016–2018) seed oil content (% of dry seed weight), protein content (% of dry seed weight) and weight/1000 dry seeds of three accessions. **b** Percentage of fatty acid species in 30SM and 30SB of three accessions (*n* = 4), 30SM and 30SB represent seeds on the main inflorescence and primary branch of 30 days after flowering, respectively. **c** FA composition in mature seeds of three accessions (mean ± SD, *n* = 4). CQ46 (Ningyou 12), CQ24 (SWU 47) and CQ52 (Zhongshuang 11) are accessions for RNA-Seq.*Denotes significance at *P* < 0.05, **P < 0.01, based on Student’s *t* test

Three natural *B. napus* accessions with extremely significant differences in seed oil content (SOC) were selected from the genome-wide association analysis populations. The oil and protein contents of the desiccated seeds were determined by near-infrared reflectance spectroscopy (NIRS DS2500), and the results are shown in Fig. 4a. The average oil contents during the 3-year period for SWU47 (CQ24, high-oil) and Zhongshuang11 (CQ52, high-oil) were significantly higher than those of Ningyou12 (CQ46, low oil) and there is no significant difference in seed protein content and 1000-seed weight between the high- and low-oil lines (Fig. 4a). To determine the FA compositions of 30SM, 30SB and mature desiccated seeds of three accessions subjected to gas chromatography–mass spectrometry (GC–MS). In 30SM, 30SB (Fig. 4b) or mature desiccated seeds (Fig. 4c), the results showed significantly higher C18:1 in CQ24 and CQ52 (high-oil content), and significantly lower C16:0 and C18:2 in CQ24 and CQ52 compared to CQ46 (low-oil content).

### Transcriptome analyses for differentially expressed genes

In the transcriptome analyses, RNA was obtained from four tissues of extremely high- (CQ24 and CQ52) and low-oil content (CQ46) *B. napus* lines at CQ including 12 independent samples and a total of 24 libraries (two biological replicates per sample) were constructed for transcriptome sequencing. After removing the low quality and contaminant reads, a total of 364.0 million clean reads were acquired, with an average of 23.05 million reads per sample. On average, 95.24% of the input reads mapped uniquely to the *B. napus* reference genome (Additional file 1: Table S2). The correlation coefficient between the two biological replicates of each sequencing sample exceeded 0.9 for all tested samples (Additional file 1: Table S2), suggesting a high reproducibility among the samples.

**Fig. 5:**
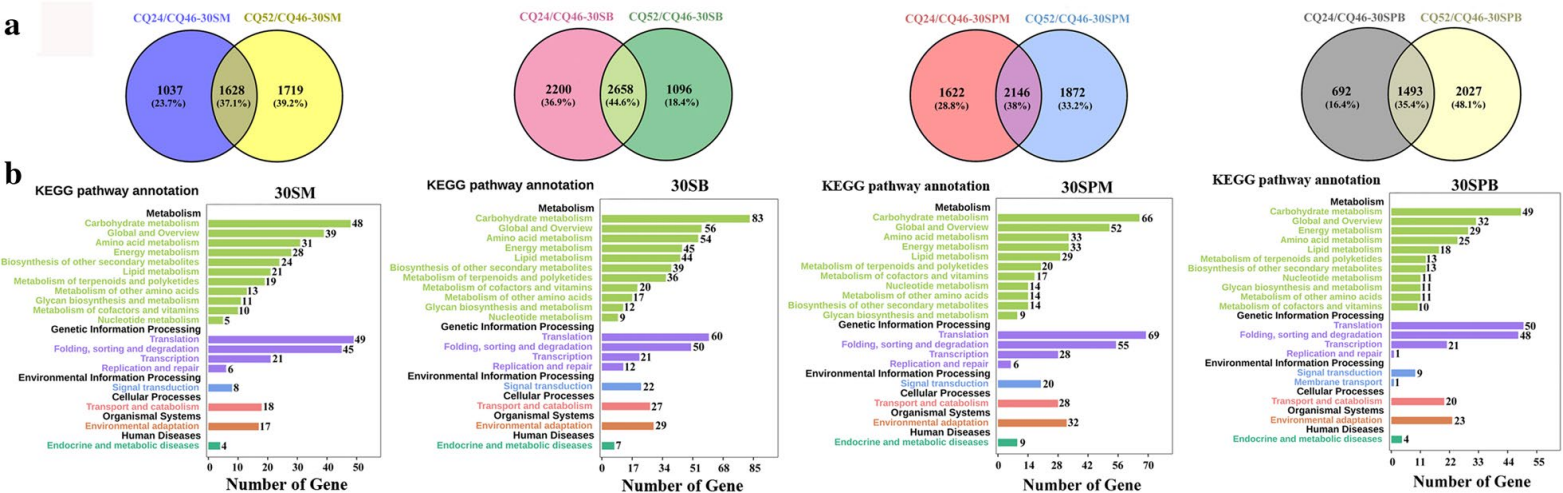
Transcriptomic analysis of the four tissues among HO and LO *B. napus* cultivars. **a** Common differential genes (DEGs) were obtained in all four tissues under CQ24/CQ46 and CQ52/CQ46 by Venny analysis. **b** KEGG pathway analysis of common DEGs in the Fig. 5a

To identify differentially expressed genes (DEGs) between extremely high- and low-oil content *B. napus* lines, the following criterion were applied: |log_2_fold change| > 2.0 and FDR < 0.05. For CQ24/CQ46, 2185 and 4858 genes were differentially expressed in individual tissues. Among which, the number of DEGs was variable, and the number of upregulated genes exceeded that of the number of downregulated genes in all tissues tested. Under CQ52/CQ46, between 3347 and 4018 genes were differentially expressed in individual tissues, among which the number of differential genes was not very variable and the number of upregulated exceeded the number of downregulated genes in all tissues tested except 30SM (Additional file 1: Table S3, Additional file 2: Fig. S1a). These results suggested that positive regulatory genes might play a major role in the formation of high oil content in *B. napus*. To find conserved DEGs between high- and low-oil content lines, Venny analysis was conducted using CQ24/CQ46 and CQ52/CQ46 in all four tissues, respectively. We found 1628, 2658, 2146 and 1493 common DEGs in 30SM, 30SB, 30SPM and 30SPB, respectively, suggesting that transcriptomic variations are different in diverse tissues and variations in 30SB and 30SPM are greater than 30SM and 30SPB between high- oil and low-oil content *B. napus* (Fig. 5a).

### Functional classification of common DEGs between CQ24 (HO)/CQ46(LO) and CQ52(HO)/CQ46(LO)

To understand the functional classification of common DEGs in four tested tissues using CQ24(HO)/CQ46(LO) and CQ52(HO)/CQ46(LO) (hereinafter referred to as common DEGs), Gene Ontology (GO) enrichment analysis was performed. GO terms were divided into three main categories: biological process, cellular component and molecular function (Additional file 2: Fig. S1b, c). The results showed that common DEGs involved in cellular (GO:0009987), metabolism (GO:0008152) and single-organism processes (GO:0044699) were the most common in all four tissues between extremely high- and low-oil content *B. napus* lines. The most common DEGs were mainly enriched in the cell part (GO:0044464), cell (GO:0005623) and organelles (GO:0043226), and the dominant molecular function of the most common DEGs in all four tissues was binding (GO:0005488) and catalytic activity (GO:0003824) (Additional file 2: Fig. S1b, c).

To understand the functional distribution of these common DEGs, Kyoto Encyclopedia of Genes and Genomes (KEGG) pathway analysis was also conducted. The results showed that the most enriched pathway was involved in metabolism and the main pathways of common DEGs in all four tested tissues were carbohydrate metabolism, global and overview, amino acid metabolism, energy metabolism, lipid metabolism and biosynthesis of other secondary metabolites (Fig. 5b). In this study, we focused on common DEGs in lipid metabolism, and 64 differential expressed lipid metabolism-related genes were found in all four tissues, of which 4, 24, 10, and 6 were specific to 30SM, 30SB, 30SPM, and 30SPB, respectively. In addition, six common differential lipid metabolism genes were shared in 30SM and 30SB, two in 30SB, 30SM and 30SPB, two in 30SB, 30SM and 30SPM, three in 30SB, 30SPB and 30SPM, and seven among all four tissues (Additional file 1: Table S4).

To understand the expression patterns of 64 common differential expressed lipid metabolism-related genes between HO (CQ24, CQ52) and LO (CQ46) accessions, the heatmap of these genes was drawn based on the RNA-seq data which were normalized to the log_2_ FPKM using HemI1.0 (Additional file 1: Table S4, Additional file 2: Fig. S2a). Among all 64 common differential expressed lipid metabolism-related genes, 38 genes were upregulated in the HO (CQ24, CQ52) lines compared with the LO (CQ46) lines, and 26 genes showed opposite expression patterns. We found seven genes (*BnaA07g31890D*; *BnaC02g06560D*; *BnaCnng62740D*; *BnaA03g60440D*; *BnaA07g35160D*; *BnaA07g20190D*; *BnaC08g44190D*) that were differentially expressed in all tested tissues between HO and LO accessions, and only *BnaC08g44190D* was downregulated in HO compared with LO. Moreover, a total of 13 DEGs (*BnaC03g16690D*; *BnaA07g31890D*; *BnaC02g00470*D; *BnaC03g04180D*; *BnaC02g04910D*; *BnaC04g40760D*; *BnaA03g13590D*; *BnaA04g17150D*; *BnaC08g12280D*; *BnaA01g15860D*; *BnaC01g18950D*; *BnaA10g09480D*; BnaC09g31660D) involved in the TAG biosynthesis pathway and TAG assembly were upregulated in the HO compared with the LO lines. Therefore, we speculated that these upregulated lipid metabolism genes in HO lines may play an indispensable role in the formation of high oil content of *B. napus*.

### TAG biosynthesis and assembly pathway gene expression in four tested tissues of the HO and LO accessions

**Fig. 6:**
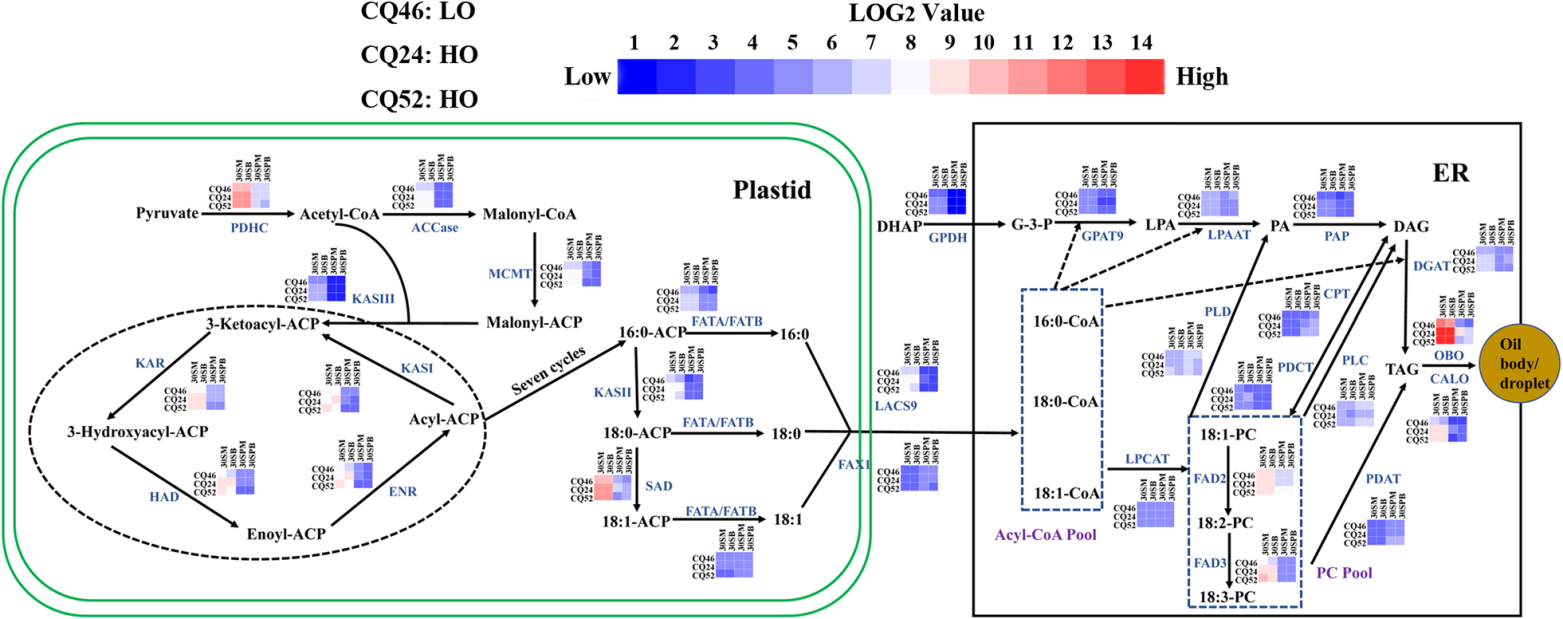
Comparison of gene expression of TAG biosynthesis pathway and TAG assembly in all four tissues among HO and LO *B. napus* lines. The color boxes are the heatmap of gene expressions from RNA-seq which were normalized by the log_2_ FPKM (calculation method) (Additional file 1: Table S6). Abbreviation of genes that encode proteins (blue letters): *PDHC* pyruvate dehydrogenase complex, *ACCase* acetyl-CoA carboxylase, *MCMT* malonyl-CoA:ACP malonyltransferase, *ACP* acyl carrier protein, *KASI/II/III* 3-ketoacyl-ACP synthase I/II/III, *KAR* ketoacyl-ACP reductase, *HAD* hydroxyacyl-ACP dehydrase, *ENR* enoyl-ACP reductase, *SAD* stearoyl-acyl carrier protein desaturase, *FATA/B* fatty acyl-ACP thioesterase A/B, *FAX1* plastid fatty acid export 1, *LACS9* long-chain acyl-CoA synthetase 9, *GPDH* glycerol-3-phosphate dehydrogenase, *GPAT9* glycerol-3-phosphate acyltransferase 9, *LPAAT* lysophosphatidic acid acyltransferase, *PAP* phosphatidic acid phosphatase, *DGAT* diacylglycerol acyltransferase, *LPCAT* lysophosphatidylcholine acyltransferase, *FAD2/3* fatty acid desaturase 2/3, *PLD* phospholipase D, *PDAT* phospholipid:diacylglycerol acyltransferase, *PDCT* phosphatidylcholine:diacylglycerol cholinephosphotransferase, *CPT* CDP-choline:diacylglycerol cholinephosphotransferase, *PLC* phospholipase C, *OBO* oil body oleosin, *CALO* caleosin

To preliminarily elucidate the difference in seed oil content between transcriptome sequencing accessions at the transcription level, the genes involved in the TAG biosynthesis pathway were analyzed and presented using a Log_2_FPKM (calculation method) as relative transcript levels among the tissues and accessions, with a specific focus on FA synthesis in the plastid, and TAG accumulation/packaging pathways in the endoplasmic reticulum (Fig. 6) [2, 34].

In terms of FA synthesis in the plastid, most genes exhibited differential expression patterns among 30SM, 30SB, 30SPM, and 30SPB tissues in the same accession, especially in seeds (30SM, 30SB), and the expression level of most FA synthesis genes was significantly higher than in silique pericarps (30SPM and 30SPB). In addition, many genes showed differential expression levels in the same tissue among the HO (CQ24, CQ52) and LO (CQ46) accessions (Fig. 6 and Additional file 1: Table S6). For example, the transcript levels of the pyruvate dehydrogenase complex (*PDHC*), acetyl-CoA carboxylase (*ACCase*), malonyl-CoA:ACP malonyltransferase (*MCMT*), 3-ketoacyl-ACP synthase II/III (*KASII/III*), ketoacyl-ACP reductase (*KAR*), hydroxyacyl-ACP dehydrase (*HAD*), enoyl-ACP reductase (*ENR*), stearoyl-acyl carrier protein desaturase (*SAD*), and acyl-ACP thioesterase A (*FATA*) were generally lower in the seed tissues (30SM and 30SB) of the LO (CQ46) than the HO (CQ24 and CQ52) accessions. However, these genes showed no difference in transcription levels in silique pericarps (30SPM and 30SPB) among the HO and LO lines. Together, the differential expression of fatty acid synthesis genes in seeds is more likely to explain the difference in oil content among the three accessions than the silique pericarps; however, this does not preclude an effect of the silique pericarps in modulating the oil content difference.

Glycerol-3-phosphate (G-3-P) and acyl-CoA, which are precursors of glycerolipid assembly in the endoplasmic reticulum (ER), are produced by NAD-dependent glycerol-3-phosphate dehydrogenase (*GPDH*), and be involved in plastid fatty acid export 1(*FAX1*) and long-chain acyl-CoA synthetase 9 (*LACS9*) [5, 12, 31]. The transcript levels of *GPDH* and *LACS9* were higher in the 30SM and 30SB of HO than in the LO lines, suggesting an increase in the acyl-CoA pool and glycerol-3-phosphate (G-3-P) substrate for HO lines in seeds. However, their expression in silique pericarps (30SPM and 30SPB) was not consistent. It is worth noting that the expression levels of *GPDH* and *LACS9* in seeds were much higher than those in silique pericarps, but the expression of *FAX1* was higher in silique pericarps than in seeds (Fig. 6). Thus, based from the transcript levels alone of the four transcriptome sequencing tissues, it remains vague whether these steps are important in regulating the oil content differences among the three accessions.

The assembly of TAGs in the ER occurs via two possible routes [9]. In the conventional Kennedy pathway, glycerol-3-phosphate (G-3-P) with acyl-CoA to yield TAG requires sequential enzymes; glycerol-3-phosphate acyltransferase 9 (*GPAT9*), 1-acylglycerol-3-phosphate acyltransferase (*LPAAT*), phosphatidic acid phosphatase (*PAP*) and diacylglycerol acyltransferase (*DGAT*) [5, 9, 12]. The transcript levels of *PAP* and *DGAT* (sum of *DGAT1* and *DGAT2*) were higher in 30SM and 30SB of HO (CQ24, CQ52) than LO (CQ46), whereas *GPAT9* and *LPAAT* exhibited similar transcript levels in 30SM and 30SB between HO (CQ24, CQ52) and LO (CQ46). In another TAG biosynthesis pathway, lysophosphatidylcholine acyltransferase (*LPCAT*) and phospholipid: diacylglycerol acyltransferase (*PDAT*) play an important role in forming TAG [9]. The transcript levels of *LPCAT* and *PDAT* were similar among the HO and LO accessions. Additionally, the transcript levels of FA desaturase 3 (*FAD3*) were higher in the seeds (30SM and 30SB) of the HO than the LO accessions, phosphatidylcholine: diacylglycerol cholinephosphotransferase (*PDCT*) and CDP-choline: diacylglycerol cholinephosphotransferase (*CPT*) could mediate the shuttling between PC-derived DAG and PC, and the transcript levels for *PDCT* were lower in seeds (30SB) of LO than HO accessions, while *CPT* transcript levels were higher in silique pericarps (30SPM and 30SPB) in the HO accessions. Phospholipase C (*PLC*) and phospholipase D (*PLD*) hydrolyze PC to produce DAG and PA, respectively, and their expression is increased in silique pericarps (30SPM and 30SPB) compared with seeds (30SM and 30SB). The oil body oleosin (*OBO*) and caleosin (*CALO*), are crucial for the stability of lipid droplets [44]. The RNA-Seq data showed that the expression level of *OBO* (sum of all isoforms) and *CALO* was lower in all tissues of the LO than HO accessions, and *OBO* had the highest expression in seeds (30SM and 30SB) of the HO accessions (Fig. 6).

### Identification candidate genes by combining GWAS and transcriptome sequencing analysis

The candidate gene regions with SOC as determined by GWAS analysis were the 300-kb flanking regions on either side of the markers significantly associated with SOC, as described previously [56]. All genes within the confidence interval of all 17 significant SNPs with SOC are listed in Additional file 1: Table S5. By combination with transcriptome sequencing analysis, we found a total of 41 genes that were differentially expressed between high and low oil accessions, of which 16 and 32 were DEGs in CQ24/CQ46 (No. X1–X16) (Table 4) and CQ52/CQ46 (NO. Z1-Z32) (Table 5), respectively. Furthermore, seven common DEGs (X5/Z30, X7/Z31, X10/Z20, X12/Z13, X16/Z10, X15/Z9, and X14/Z1) were identified between CQ24/CQ46 and CQ52/CQ46. The biological functions of these differential expressed candidates were analyzed by applying Protein Basic Local Alignment Search Tool (BLASTP) searches against all *Arabidopsis* proteins. The results are listed in Tables 4 and 5 and the expression patterns of these genes between high (CQ24, CQ52) and low oil (CQ46) accessions are shown in Additional file 2: Fig. S3. Seven common DEGs (X5/Z30, X7/Z31, X10/Z20, X12/Z13, X16/Z10, X15/Z9, and X14/Z1) were considered to be important candidate genes related to seed oil content for the following study.

### Verification of the transcriptome sequencing data by quantitative real-time polymerase chain reaction (qRT-PCR)

**Fig. 7:**
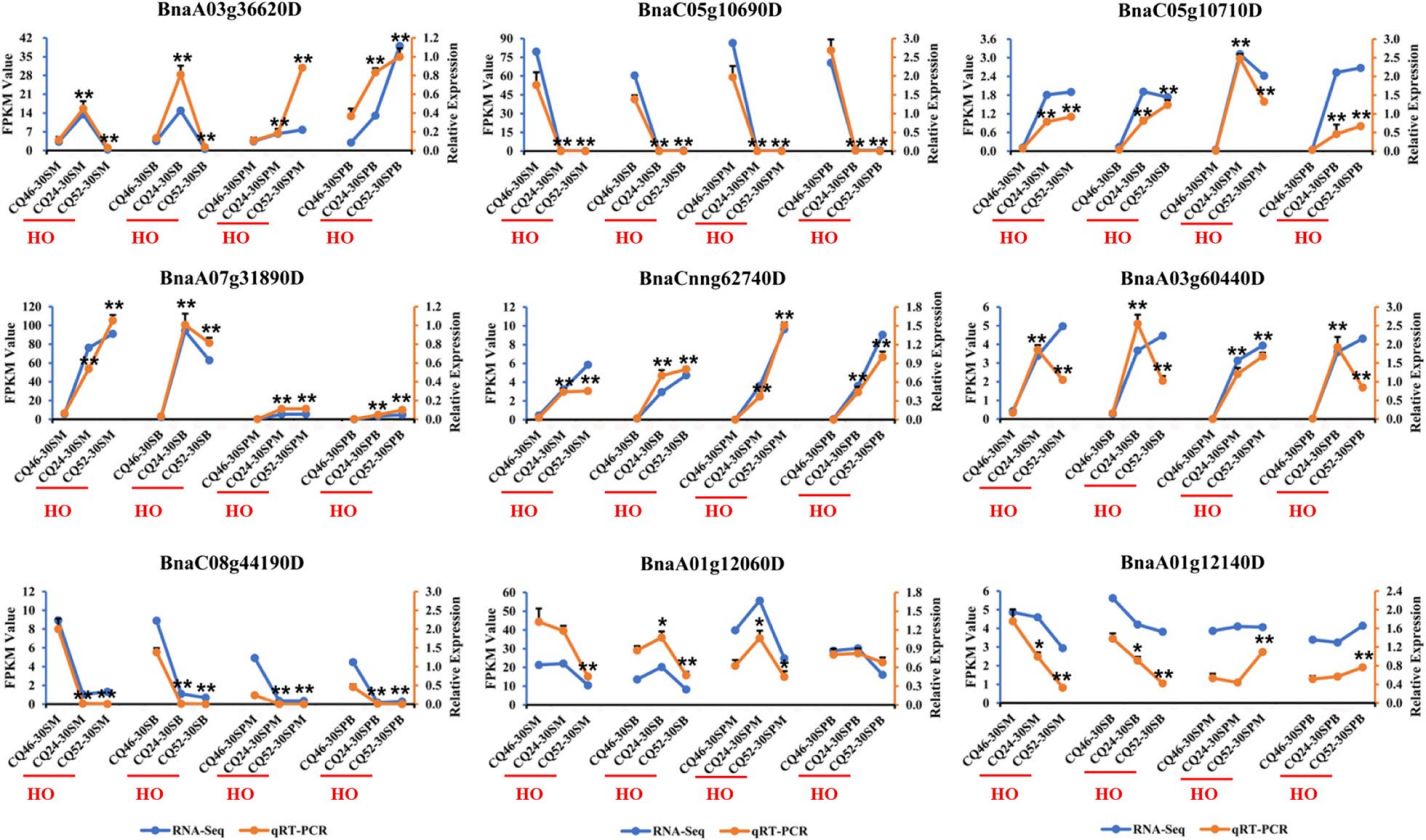
qRT-PCR analysis confirmed the accuracy of transcriptome sequencing by identifying the expression patterns of nine genes in all four tested tissues. The blue line represents the RNA-Seq results and the orange line represents the qRT-PCR results. *Denotes significance at *P* < 0.05, **P < 0.01, based on Student’s *t* test

To confirm the accuracy of the RNA-Seq results, some genes were selected to perform qRT-PCR analysis, and the expression levels of these genes by qRT-PCR and transcriptome sequencing are shown in Fig. 7. Although the expression trend of individual genes, such as *BnaA03g60440D*, was not consistent with the RNA-Seq results between two high-oil content accessions, their expression trend was consistent between high- and low-oil content accessions. In short, the results of RNA-Seq are highly consistent with qRT-PCR. The results fully demonstrate the reliability and accuracy of the transcriptome sequencing data.

## Discussion

### The identification of significant SNPs for seed oil content (SOC)

Genome-wide association analysis (GWAS) based on millions of markers is currently widely used in analysis of complex agronomic traits for crops such as rice [22], maize [50], soybean [23] and sorghum [46]. Many reports have addressed QTL mapping of seed oil content in *Brassica napus* [8, 10, 13, 24, 49, 54, 55, 59, 68]. However, there have been relatively few reports on the identification of significant SNPs associated with seed oil content by GWAS compared with the QTL mapping method. Liu et al. identified 50 SNPs that were significantly associated with seed oil content using 521 *B. napus* accessions combined with the *Brassica* 60K SNP array [36]. Seven stable QTLs for seed oil content were identified by Sun et al. by combined linkage and association mapping methods in *Brassica napus* [51]. Four significant SNPs for seed oil content were located on chromosomes A1, C3, and C5 [27]. Seventeen loci associated with seed oil content were identified by combining high-throughput genome resequencing and phenotyping using GWAS [56]. Although previous studies have identified many SNPs that were significantly associated with SOC in *Brassica napus*, none of them have combined the analysis with transcriptome sequencing to obtain candidate genes contributing to SOC. In our previous study, the population structure and linkage disequilibrium of 588 *Brassica napus* accessions were evaluated, and provided a high-resolution genomic variation map consisting of 616053 SNPs by the resequencing method. In the present study, we obtained 17 SNPs that were significantly associated with seed oil content (SOC) in *Brassica napus*, 12 of which were overlapped with previous studies (Table 2), supporting the high reliability of loci detected in current study. In addition, we also found five new SNPs significantly associated with SOC in *Brassica napus* (Table 2), and all the significant SNPs detected in current study were further analyzed by combination with the RNA-Seq data.

### Comparative analysis of three *Brassica napus* lines using transcriptome sequencing from phenotype to gene expression

Identification of differentially expressed (DE) candidate genes in combination with GWAS and RNA-seq has been proven to be more successful than each separate strategy [38, 64]. In this study, an extremely low-oil (CQ46) and two extremely high-oil content accessions (CQ24, CQ52) were selected from the GWAS analysis population to perform transcriptome sequencing (Fig. 4). A difference of approximately 10% SOC was observed between the HO and LO accessions (Fig. 4a), without a difference between the seed protein content and 1000-grain weight. Additionally, C18:1 was higher in HO (CQ24 and CQ52) than LO (CQ46) accessions, and C16:0 and C18:2 showed the opposite pattern either in seeds at 30 days after flowering or at maturity, which are consistent with the higher expression levels of *KASIII*, *SAD* and *FAD3* in seeds (30SM and 30SB) of high-oil content than low-oil content accessions (Fig. 6). Moreover, a previous study has shown that fatty acid accumulation is accelerated from 26 to 34 days after flowering [40]. Therefore, seeds and silique pericarps at 30 days after flowering were selected for transcriptome analysis among typical high- and low-oil content accessions. In addition, we analyzed the transcriptional levels of major genes in the TAG biosynthesis and assembly pathway in four tissues of HO (CQ24, CQ52) and LO (CQ46) accessions. As shown in Fig. 6, transcripts of the genes involved in the TAG biosynthesis and assembly pathway were generally higher in seeds (30SM and 30SB) than silique pericarps (30SPM and 30SPB), which suggested that the seeds were more active in TAG biosynthesis and assembly than silique pericarps. Exceptionally, we found that *FAX1*, *PLD*, *CPT*, *PLC*, and *PDAT* were expressed at higher levels in silique pericarps than seeds, indicating that silique pericarps may also play a non-negligible role in TAG biosynthesis, which is consistent with a previous study examining the importance of the silique wall in the regulation of seed oil content [18]. The expression level of most of genes involved in fatty acid (FA) and TAG biosynthesis in seeds (30SM and 30SB) was significantly enhanced in HO (CQ24 and CQ52) compared with LO, which including *PDHC*, *ACCase*, *MCMT*, *KAR*, *HAD*, *KASIII*, *FATA*, *KASII*, *SAD*, *LACS9*, *GPDH*, *PAP*, *FAD3*, *DGAT* and so on (Fig. 6, Additional file 1: Table S6). To our surprise, the transcription levels of *OBO* and *CALO* were significantly higher in all tested tissues of HO (CQ24 and CQ52) than LO (CQ46) (Fig. 6, Additional file 1: Table S6), which may suggest an important role for these *OBO* and *CALO* in high seed oil formation. This result is consistent with the findings of Liu et al. showing that the overexpression of soybean oleosin can increase the seed lipid content in transgenic rice [35]. These results strongly suggested that it is feasible to select these accessions as extremely high- (CQ24 and CQ52) and low-oil content (CQ46) lines for RNA-Seq.

### The identification of SOC-related candidate genes

*Brassica napus* is one of the most important oil crops in the world along with soybean and palm. The typical SOC of *B. napus* germplasm varies from 35 to 50% [36]. Recently, through the efforts of breeders, ultra-high oil content germplasm materials with 55–65% oil content have been produced [20]. Hu et al. predicted that the *B. napus* seed oil content could be increased to 75% [17]. Therefore, there is still a great potential to increase the *B. napus* seed oil content, especially in the main Chinese rapeseed producing areas such as Chongqing of the Yangtze River Basin. Previous studies examining the seed oil content of *B. napus* have focused on many QTL mappings or a small number of GWAS, but in this study, the combination of GWAS and transcriptome sequencing was implemented for this purpose. According to the 300-kb flanking regions on either side of the 17 significantly associated SNPs with SOC [56], we obtained a total of 411 genes. These genes were annotated by applying the BLASTP program against the *Arabidopsis* proteome (TAIR10) with an *E*-value threshold of 1E−5 [38] (Additional file 1: Table S5), and a total of 14 acyl-lipid metabolism (ALM)-related genes were found (Table 3). Although these 14 genes did not differ significantly in the four detested tissues of high- and low-oil accessions, and their expression patterns are shown in Additional file 2: Fig. S2b, we found *BnaC05g10520*, *BnaA01g12060* and *BnaA01g12140D* were expressed at higher levels in all tested tissues of high- and low-oil accessions. In previous reports, *BnaA01g12140D* which is homologous to *AT4G22330* (*ATCES1*), encodes a nuclear and endoplasmic reticulum localized Acyl-CoA-independent ceramide synthase that is involved in sphingolipid metabolism, disease resistance, nutrient limitation, and response to salt stress [62, 67]. Therefore, we speculate that these genes (*BnaC05g10520*, *BnaA01g12060* and *BnaA01g12140D*) play an important role in lipid metabolism of *B. napus*.

**Table 3:** All genes within the confidence interval of significant related SNPs with SOC belong to ALM genes

ALM genes in confidence interval	At orthologs	Pathways	Function description
BnaC05g10520D	AT1G14290	Sphingolipid biosynthesis 1	Sphingoid base hydroxylase 2 (SBH2)
BnaA01g12830D	AT1G73550	Fatty acid elongation and wax biosynthesis	Bifunctional inhibitor/lipid-transfer protein/seed storage 2S albumin superfamily protein
BnaC07g34330D	AT3G10550	Phospholipid signaling	Myotubularin-like phosphatases II superfamily
BnaA03g36710D	AT3G22620	Fatty acid elongation and wax biosynthesis	Bifunctional inhibitor/lipid-transfer protein/seed storage 2S albumin superfamily protein
BnaA03g37090D	AT3G23530	Unknown	Cyclopropane-fatty-acyl-phospholipid synthase
BnaA03g36540D	AT4G11850	Phospholipid signaling	Phospholipase D gamma 1 (PLDGAMMA1)
BnaC07g33970D	AT4G16820	Prokaryotic galactolipid, sulfolipid, and phospholipid synthesis 2	Alpha/beta-hydrolases superfamily protein
BnaC07g34360D	AT4G17483	Unknown	Alpha/beta-hydrolases superfamily protein
BnaA01g12060D	AT4G22240	Unknown	Plastid-lipid associated protein PAP/fibrillin family protein
BnaA01g12140D	AT4G22330	Sphingolipid biosynthesis 2	ATCES1
BnaA01g12150D	AT4G22340	Prokaryotic galactolipid, sulfolipid, and phospholipid synthesis 1	Cytidinediphosphate diacylglycerol synthase 2 (CDS2)
BnaA01g12260D	AT4G22520	Fatty acid elongation and wax biosynthesis	Bifunctional inhibitor/lipid-transfer protein/seed storage 2S albumin superfamily protein
BnaA01g12290D	AT4G22550	Prokaryotic galactolipid, sulfolipid, and phospholipid synthesis 1	Phosphatidic acid phosphatase (PAP2) family protein
BnaA01g12350D	AT4G22640	Fatty acid elongation and wax biosynthesis	Bifunctional inhibitor/lipid-transfer protein/seed storage 2S albumin superfamily protein

A combination of our GWAS and the DEGs of the transcriptome sequencing results between HO and LO accessions revealed 16 genes (X1–X16) under CQ24/CQ46 (Table 4) and 32 genes (Z1–Z32) under CQ52/CQ46 (Table 5). The expression patterns of all the genes using HemI1.0 are shown in Additional file 2: Fig. S3 [58]. Interestingly, seven common genes were obtained under CQ24/CQ46 and CQ52/CQ46, and they were considered important candidate genes associated with seed oil content in *B. napus*. *BnaA01g13030D* (X5/Z30) and *BnaA03g36510D* (X7/Z31) represented two genes of unknown function, and their expression levels were significantly lower in seeds of HO (CQ24 and CQ52) than LO (CQ46) (Additional file 2: Fig. S3c). The gene (BnaA03g36620D, X10/Z20) encodes AOX1a, an isoform of alternative oxidase. The absence of AOX1a in *Arabidopsis* leads to acute sensitivity to combined light and drought stress [15]. AOX1a has been implicated in the modulation of metabolic homeostasis in cadmium (Cd)-exposed *Arabidopsis* plant and displays a differential role in roots and leaves in response to sublethal cadmium exposure [25, 26]. The maize AOX1a gene plays an essential role under oxidative stress [47]. In *Brassica napus* roots, selenite can activate the alternative oxidase pathway and alter primary metabolism and may ultimately improve selenium tolerance [11]. *BnaA03g37060D* (X12/Z13) was a putative component of photosystem II complex and may be involved in response to UV-B, ozone and wounding. In all tested tissues, *BnaA03g37060D* is less expressed in HO and is not expressed in LO accessions. *BnaC05g10690D* (X14/Z1) exhibited extremely high expression levels in LO compared with HO lines, and this finding was verified by qRT-PCR (Fig. 7, Additional file 2: Fig. S3c). Therefore, we speculate that interfering with the expression of this gene may contribute to increase seed oil content in *Brassica napus*. *BnaC05g10700D* (X15/Z9) was an ARM repeat superfamily protein and its specific function is unknown, and its expression level in HO is higher than that of LO accessions. Increasing the expression level of *BnaC05g10700D* may help to increase seed oil content. BON2 (BnaC05g10710D, X16/Z10) encodes a copine-like protein. In *Arabidopsis*, the *BON* gene family can promote cell growth and development in addition to repressing cell death [63]. Additionally, Li et al. [33] have validated that plasma membrane-localized calcium pumps and copines coordinately regulate pollen germination and fertility in *Arabidopsis*. Furthermore, *BnaC05g10710D* exhibited a significantly higher expression level in HO compared with LO accessions, and this result also further verified by qRT-PCR (Fig. 7). This gene (*BnaC05g10710D*) may play a positive regulatory role in the formation of high oil content in *B. napus*. Of course, the role of the seven candidate genes mentioned above in the formation of seed oil content in *B. napus* must be further confirmed, which will be an important task for our next studies.

**Table 4:** Candidate genes screened by combining GWAS and RNA sequencing (CQ24/46)

Code	Candidate genes	At orthologs	Location	gDNA Length (bp)	CDS length (bp)	Exon number	Function description
X1	BnaA01g11960D	AT4G22160	chrA01:6001930–6002661	732	480	2	Unknown protein
X2	BnaA01g12400D	AT4G22730	chrA01:6194675–6197498	2824	2064	3	Leucine-rich repeat protein kinase family protein
X3	BnaA01g12530D	AT4G22880	chrA01:6294325–6295940	1616	1077	2	Leucoanthocyanidin dioxygenase (LDOX)
X4	BnaA01g12540D	AT4G22890	chrA01:6297451–6299173	1723	960	9	PGR5-LIKE A
X5	BnaA01g13030D	AT4G23370	chrA01:6518736–6520647	1912	1161	8	Unknown protein
X6	BnaA03g36210D	AT3G21320	chrA03:17700381–17702480	2100	1473	5	EARLY FLOWERING protein
X7	BnaA03g36510D	AT3G29040	chrA03:17886150–17887585	1436	744	3	Domain of unknown function (DUF26)
X8	BnaA03g36520D	AT3G22160	chrA03:17918805–17921792	2988	264	2	JASMONATE-ASSOCIATED VQ MOTIF GENE 1, JAV1
X9	BnaA03g36540D	AT4G11850	chrA03:17925272–17933870	8599	4398	15	Phospholipase D gamma 1 (PLDGAMMA1)
X10	BnaA03g36620D	AT3G22370	chrA03:17988398–17992403	4006	1065	6	Alternative oxidase 1A (AOX1A)
X11	BnaA03g37000D	AT3G23180	chrA03:18246929–18248163	1235	564	6	HR-like lesion-inducing protein-related
X12	BnaA03g37060D	AT1G51400	chrA03:18277785–18278767	983	438	3	Photosystem II 5 kD protein
X13	BnaC05g10400D	AT1G14130	chrC05:5962733–5963804	1072	915	2	2-oxoglutarate (2OG) and Fe(II)-dependent oxygenase superfamily protein
X14	BnaC05g10690D	AT1G14450	chrC05:6142756–6148159	5404	225	1	NADH dehydrogenase (ubiquinone)s
X15	BnaC05g10700D	AT1G51350	chrC05:6148823–6151086	2264	312	7	ARM repeat superfamily protein
X16	BnaC05g10710D	AT5G07300	chrC05:6151155–6152461	1307	357	8	Encodes a copine-like protein, which is a member of a newly identified class of calcium-dependent, phospholipid binding proteins

**Table 5:** Candidate genes screened by combining GWAS and RNA sequencing (CQ52/46)

Code	Genes in confidence interval	At orthologs	Location	gDNA length (bp)	CDS length (bp)	Exon number	Function description
Z1	BnaC05g10690D	AT1G14450	chrC05:6142756–6148159	5404	225	1	NADH dehydrogenase (ubiquinone)s
Z2	BnaA03g37070D	AT3G23410	chrA03:18280209–18283825	3617	2166	3	Fatty alcohol oxidase 3 (FAO3)
Z3	BnaA01g12270D	AT4G22530	chrA01:6118512–6120375	1864	786	2	*S*-Adenosyl-l-methionine-dependent methyltransferases superfamily protein
Z4	BnaA01g12520D	AT4G22860	chrA01:6288006–6294204	6199	2358	23	Cell cycle regulated microtubule-associated protein
Z5	BnaA01g12870D	AT4G23060	chrA01:6443916–6447139	3224	1467	5	IQ-domain 22 (IQD22)
Z6	BnaA03g36230D	AT3G21330	chrA03:17713723–17714730	1008	1008	1	Basic helix-loop-helix (bHLH) DNA-binding superfamily protein
Z7	BnaA03g36910D	AT3G22960	chrA03:18136808–18141526	4719	1728	7	PKP-ALPHA
Z8	BnaC07g34230D	AT4G17220	chrC07:37116980–37119308	2329	1512	10	Microtubule-associated proteins 70-5 (MAP70-5)
Z9	BnaC05g10700D	AT1G51350	chrC05:6148823–6151086	2264	312	7	ARM repeat superfamily protein
Z10	BnaC05g10710D	AT5G07300	chrC05:6151155–6152461	1307	357	8	Putative copine, regulates calcium signalling, BONZAI (AtBON2)
Z11	BnaC07g34010D	AT4G16980	chrC07:36973813–36974752	940	513	1	Arabinogalactan-protein family
Z12	BnaC07g34060D	AT4G17050	chrC07:37001028–37003893	2866	903	13	Ureidoglycine aminohydrolase (UGLYAH)
Z13	BnaA03g37060D	AT1G51400	chrA03:18277785–18278767	983	438	3	Photosystem II 5 kD protein
Z14	BnaA01g12040D	AT4G22220	chrA01:6038697–6039866	1170	480	3	ISU1
Z15	BnaA01g12370D	AT4G22680	chrA01:6178215–6179541	1327	804	2	Myb domain protein 85 (MYB85)
Z16	BnaC07g34050D	AT4G17040	chrC07:36997388–37000753	3366	921	7	CLP protease R subunit 4 (CLPR4)
Z17	BnaA01g12340D	AT4G22620	chrA01:6165884–6166560	677	483	1	SAUR-like auxin-responsive protein family
Z18	BnaC05g10380D	AT1G14100	chrC05:5938383–5940030	1648	1578	2	Fucosyltransferase 8 (FUT8)
Z19	BnaA03g36300D	AT3G21460	chrA03:17739884–17740442	559	309	1	Glutaredoxin family protein
Z20	BnaA03g36620D	AT3G22370	chrA03:17988398–17992403	4006	1065	6	Alternative oxidase 1A (AOX1A)
Z21	BnaA01g12320D	AT4G22590	chrA01:6150232–6152082	1851	1128	7	Haloacid dehalogenase-like hydrolase (HAD) superfamily protein
Z22	BnaA01g12930D	AT4G23180	chrA01:6474955–6477798	2844	2007	7	Cysteine-rich RLK (RECEPTOR-like protein kinase) 10 (CRK10)
Z23	BnaC05g10460D	AT1G14200	chrC05:6006999–6010819	3821	1059	4	RING/U-box superfamily protein
Z24	BnaC07g34250D	AT4G17260	chrC07:37125045–37126398	1354	1053	2	Lactate/malate dehydrogenase family protein
Z25	BnaC07g34450D	AT4G17615	chrC07:37207760–37210093	2334	642	8	Calcineurin B-like protein 1 (CBL1)
Z26	BnaC07g34510D	AT4G17670	chrC07:37221343–37222462	1120	507	2	Protein of unknown function (DUF581)
Z27	BnaA03g36680D	AT4G30090	chrA03:18023844–18026170	2327	831	10	Embryo defective 1353 (emb1353)
Z28	BnaC05g10680D	AT1G14440	chrC05:6131908–6132828	921	921	1	Homeobox protein 31 (HB31)
Z29	BnaA01g11920D	AT4G22120	chrA01:5970386–5973641	3256	2088	8	ERD (early-responsive to dehydration stress) family protein
Z30	BnaA01g13030D	AT4G23370	chrA01:6518736–6520647	1912	1161	8	Unknown protein
Z31	BnaA03g36510D	AT3G29040	chrA03:17886150–17887585	1436	744	3	Domain of unknown function (DUF26)
Z32	BnaC05g10510D	AT1G14280	chrC05:6028460–6029815	1356	1254	1	Phytochrome kinase substrate 2 (PKS2)

## Conclusion

In the present study, 17 loci significantly associated with seed oil content in *B. napus* were successfully obtained, and 12 significant SNPs were found to overlap with QTLs from previous studies, which proved the reliability of this study. In addition, five novel significant SNPs distributed on the C5 and C7 chromosomes were identified, which provided valuable information for further exploration of genes that contribute to increase seed oil content in *B. napus*. Subsequently, the combination of GWAS and transcriptome analyses revealed seven functional candidate genes located within the confidence intervals of significant SNPs associated with seed oil content in *B. napus*. These results may facilitate marker-based breeding for higher seed oil content in *B. napus*.

## Supplementary information

**Additional file 1: Table S1.** Seed oil content (SOC, % of seed weight) phenotypes of 588 accessions for GWAS analysis. 2016CQ, 2017CQ and 2018CQ refer to the three environments, CQ refers to Chongqing. BLUP represents the SOC phenotypic value obtained by the best linear unbiased prediction in three environments. Missing data are replaced by ‘− 999’. **Table S2.** The quality statistics of RNA sequencing data. **Table S3.** The number of differential genes is counted under HO/LO accessions. **Table S4.** Common differential lipid metabolism genes in four tested tissues under CQ24/CQ46 and CQ52/CQ46 based on KEGG pathway analysis. **Table S5.** All genes within the confidence interval of significant related SNPs with SOC. **Table S6.** Summary of gene expression of TAG biosynthesis and assembly pathway in all four tissues among LO (CQ46) and HO (CQ24, CQ52) *Brassica napus* lines. **Table S7.** Primer sequences for qRT-PCR verification in this study.

**Additional file 2: Fig. S1.** (a) Statistics on the number of differential genes in different tissues with different seed oil content (SOC) *Brassica napus* lines. (b) Gene ontology (GO) enrichment analysis of common DEGs in 30SM and 30SB in Fig. 5a. (c) Gene ontology (GO) enrichment analysis of common DEGs in 30SPM and 30SPB in Fig. 5a. **Fig. S2.** Expression patterns of identified ALM genes within the confidence interval of significant related SNPs with SOC. (a) Heatmap of identified common differential ALM genes was derived from KEGG pathway analysis in all tested tissues under CQ24/CQ46 and CQ52/CQ46. (b) Heat map of identified all ALM genes within the confidence interval significantly associated with SOC was derived from transcriptome sequencing among HO (CQ24, CQ52) and LO (CQ46) lines. **Fig. S3.** Expression patterns of candidate genes identified by GWAS and transcriptome sequencing. Heatmap of identified candidate genes was derived from RNA sequencing data in all tested tissues between CQ24/CQ46 (a) and CQ52/46 (b) and common candidate genes (c).

## Data Availability

All data generated or analyzed during this study are included in this published article and its additional files

## Abbreviations

GWAS: Genome-wide association study
SNP: Single nucleotide polymorphism
MAF: Minor allele frequency
DEGs: Differentially expressed genes
HO: High-oil content accessions
LO: Low-oil content accessions
TAG: Triacylglycerol
ER: Endoplasmic reticulum
SOC: Seed oil content
QTL: Quantitative trait locus
qRT-PCR: Quantitative real-time PCR
BLUP: Best linear unbiased prediction
30SM: Seeds on the main inflorescence after flowering 30 days
30SPM: Silique pericarps on the main inflorescence after flowering 30 days
30SB: Seed on the primary branch after flowering 30 days
30SPB: Silique pericarps on the primary branch after flowering 30 days
RNA-seq: RNA sequencing
FPKM: Fragments per kilobase of exon per million reads mapped
FDR: False discovery rate
ALM: Acyl-lipid metabolism
CV: Coefficient of variation
GC–MS: Gas chromatography–mass spectrometry
KEGG: Kyoto encyclopedia of genes and genomes
FA: Fatty acid
PDHC: Pyruvate dehydrogenase complex
ACCase: Acetyl-CoA carboxylase
MCMT: Malonyl-CoA:ACP malonyltransferase
ACP: Acyl carrier protein
KASI/II/III: 3-Ketoacyl-ACP synthase I/II/III
KAR: Ketoacyl-ACP reductase
HAD: Hydroxyacyl-ACP dehydrase
ENR: Enoyl-ACP reductase
SAD: Stearoyl-acyl carrier protein desaturase
FATA/B: Acyl-ACP thioesterase A/B
G-3-P: Glycerol-3-phosphate
GPDH: Glycerol-3-phosphate dehydrogenase
FAX1: Fatty acid export 1
LACS9: Long-chain acyl-CoA synthetase 9
GPAT9: Glycerol-3-phosphate acyltransferase 9
LPAAT: Lysophosphatidic acid acyltransferase
PAP: Phosphatidic acid phosphatase
DGAT: Diacylglycerol acyltransferase
LPCAT: Lysophosphatidylcholine acyltransferase
PDAT: Phospholipid:diacylglycerol acyltransferase
FAD2: FA desaturase 2
FAD3: FA desaturase 3
PDCT: Phosphatidylcholine:diacylglycerol cholinephosphotransferase
CPT: CDP-choline:diacylglycerol cholinephosphotransferase
PLC: Phospholipase C
PLD: Phospholipase D
OBO: Oil body oleosin
CALO: Caleosin
