# Supplementary material for: Identification of candidate genes controlling oil content by combination of genome-wide association and transcriptome analysis in the oilseed crop Brassica napus
Source: Biotechnol Biofuels. 2019 Sep 10;12:216. doi: 10.1186/s13068-019-1557-x (PMC6737612; doi:10.1186/s13068-019-1557-x)
Supplement: Supplementary file 2 — Additional file 2: Fig. S1. (a) Statistics on the number of differential genes in different tissues with different seed oil content (SOC) Brassica napus lines. (b) Gene ontology (GO) enrichment analysis of common DEGs in 30SM and 30SB in Fig. 5a. (c) Gene ontology (GO) enrichment analysis of common DEGs in 30SPM and 30SPB in Fig. 5a. Fig. S2. Expression patterns of identified ALM genes within the confidence interval of significant related SNPs with SOC. (a) Heatmap of identified common differential ALM genes was derived from KEGG pathway analysis in all tested tissues under CQ24/CQ46 and CQ52/CQ46. (b) Heat map of identified all ALM genes within the confidence interval significantly associated with SOC was derived from transcriptome sequencing among HO (CQ24, CQ52) and LO (CQ46) lines. Fig. S3. Expression patterns of candidate genes identified by GWAS and transcriptome sequencing. Heatmap of identified candidate genes was derived from RNA sequencing data in all tested tissues between CQ24/CQ46 (a) and CQ52/46 (b) and common candidate genes (c). [file 13068_2019_1557_MOESM2_ESM.docx]

**Additional figures: 3**

**
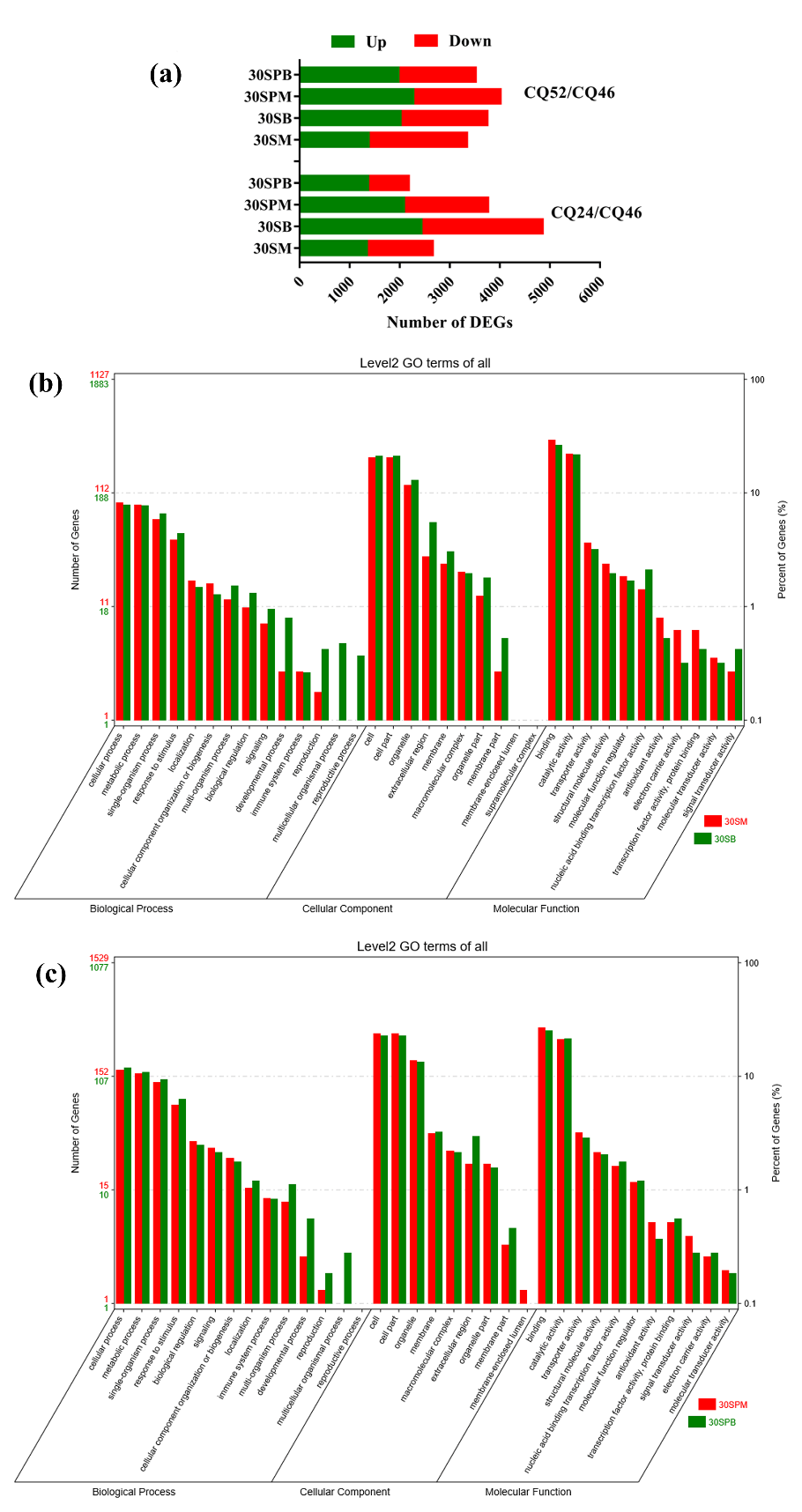
**

**Fig. S1.** Transcriptomic analysis of the HO and LO cultivars. (a) Statistics on the number of differential genes in different tissues with different seed oil content (SOC) Brassica napus lines. (b) Gene Ontology (GO) enrichment analysis of common DEGs.


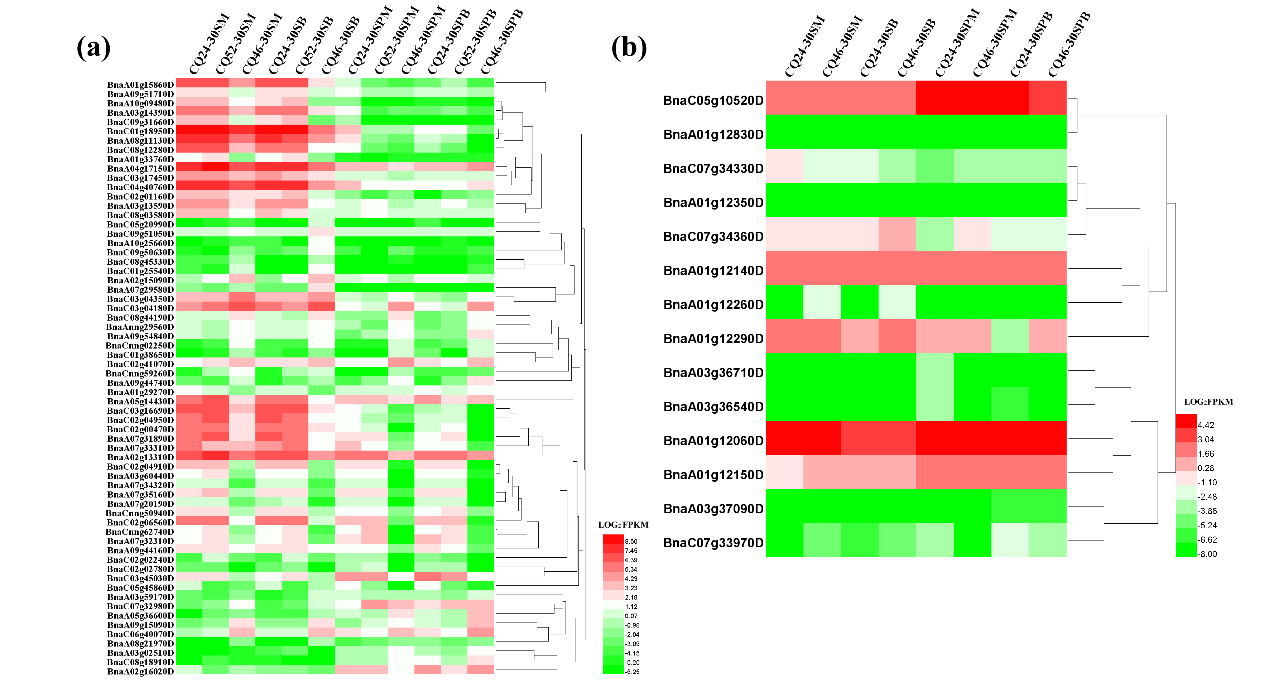


**Fig. S2** Expression patterns of identified ALM genes within the confidence interval of significant related SNPs with SOC. (a) Heatmap of identified common differential ALM genes was derived from KEGG pathway analysis in all tested tissues under CQ24/CQ46 and CQ52/CQ46. (b) Heat map of identified all ALM genes within the confidence interval significantly associated with SOC was derived from transcriptome sequencing among HO and LO lines

**
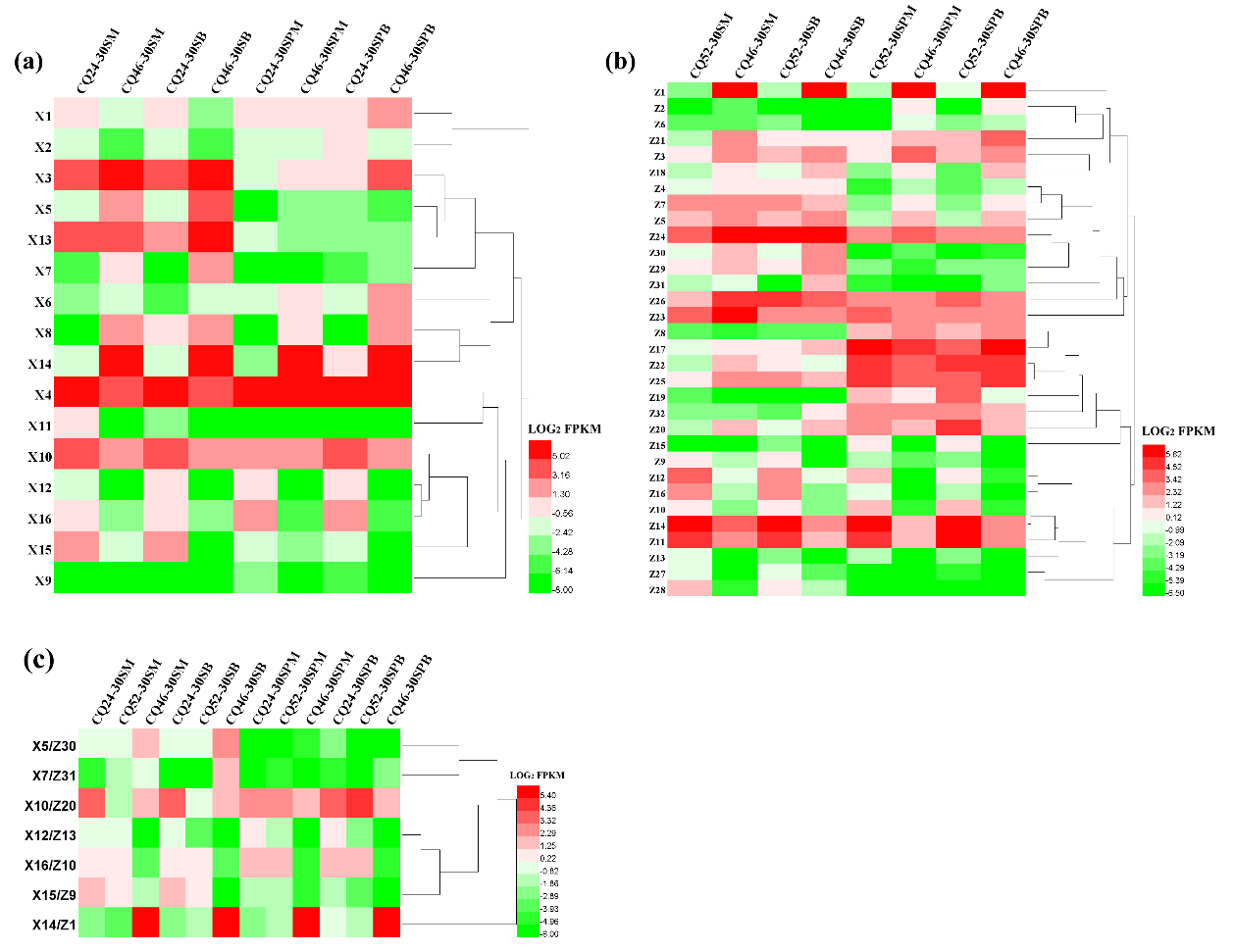
**

**Fig. S3** Expression patterns of candidate genes identiﬁed by GWAS and transcriptome sequencing. Heatmap of identified candidate genes was derived from RNA sequencing data in all tested tissues between CQ24/CQ46 (a) and CQ52/46 (b) and common candidate genes (c)
